# Association of macular structure, function, and vessel density with foveal threshold in advanced glaucoma

**DOI:** 10.1038/s41598-022-24129-1

**Published:** 2022-11-17

**Authors:** Woo Keun Song, Ko Eun Kim, Joo Young Yoon, Anna Lee, Michael S. Kook

**Affiliations:** Department of Ophthalmology, College of Medicine, Asan Medical Center, University of Ulsan, 88, Olympic-ro 43-gil, Songpa-gu, Seoul, 05505 Korea

**Keywords:** Eye diseases, Visual system

## Abstract

Identifying new biomarkers associated with central visual function impairment is important in advanced glaucoma patients. This retrospective cross-sectional study enrolled 154 eyes from 154 subjects, consisting of 86 patients with advanced open-angle glaucoma (mean deviation of 24-2 visual field [VF] tests < − 15 dB) and 68 healthy controls. Structure, function, and vessel density (VD) parameters were obtained using optical coherence tomography (OCT), 24-2 standard automated perimetry, and OCT angiography, respectively. The relationships of macular thickness, central 5° and 10° VF mean sensitivity (MS), and macular VD parameters with foveal threshold (FT), representing central visual function, were investigated using partial correlation analyses and linear regression analyses, with age adjustment. Superficial and deep layer macular VD, central 5° and 10° VF MS, and best corrected visual acuity (BCVA) correlated significantly with FT after age adjustment (*P* < 0.05). In multivariate linear regression analyses, FT associated significantly with BCVA (β = − 8.80, *P* < 0.001), central 5° MS (β = 0.30, *P* = 0.037), and deep-layer global parafoveal VD (β = 0.37, *P* = 0.037). Thus, deep-layer parafoveal VD is an independent predictor of FT and may be a potential biomarker for central visual function in advanced glaucoma.

## Introduction

Glaucomatous optic neuropathy is defined as progressive loss of retinal ganglion cells or retinal nerve fiber layer (RNFL) atrophy with subsequent visual field (VF) defects, which lead to functional impairment^1^. In early-to-moderate stage glaucoma, a combination of structural and VF tests is used to monitor disease progression^2,3^. However, disease surveillance in advanced glaucoma frequently relies upon functional tests, such as VF 24-2 or VF 10-2 tests, due to the “floor effect” of the structural tests and poor structure–function relationship^4–6^. While VF 24-2 is routinely used to monitor glaucoma, it is less optimal in eyes with advanced glaucoma due to a lack of detailed spatial information in the central 10° VF area and large test-to-retest variability^6^. While the VF 10-2 program is more sensitive in detecting VF loss and functional progression in advanced glaucoma^7^, it has the drawbacks of a lengthy test time and reduced test reproducibility^8,9^. Therefore, there is a need for an alternative functional test that can objectively estimate and monitor central visual function (CVF) at an advanced stage of glaucoma.

Foveal threshold (FT), which can be readily acquired in 15 s at the beginning of the 24-2 VF test using a Humphrey field analyzer, is closely associated with visual acuity (VA)^10,11^. Our group has recently shown that FT correlates well with CVF indicators (e.g., VA or central 5° VF mean sensitivity [MS]) as well as macular thickness and vessel density (VD) obtained using optical coherence tomography angiography (OCT-A) in early-to-moderate stage glaucoma subjects, indicating that FT may represent CVF^12^. FT measurement may include the following advantages: First, FT provides a more objective estimation of CVF than does VA in advanced glaucoma patients with severely impaired VA (e.g., VA < 20/200 or counting fingers), as it is based on a full threshold-testing algorithm^13^. Second, FT measurement may be more reproducible, as it is conducted in a more controlled environment than VA measurement.

Macular VD derived from OCT-A is diminished and is significantly associated with the severity of VF loss in glaucoma^14,15^. In addition to cross-sectional vasculature-function relationship, lower macular VD was associated with the faster CVF progression, indicating that macular VD is crucial for the detection of CVF assessment and its progression^16,17^. Furthermore, macular VD correlates better with VA than do macular thickness parameters measured with optical coherence tomography (OCT) in advanced glaucoma^6^. Therefore, we hypothesized that macular VD may serve as a surrogate marker for CVF in advanced glaucoma, since it has a lower measurement floor and a better correlation with VF sensitivity than do structural parameters (e.g., RNFL or macular thickness) in advanced glaucoma^5,14,15,18^. With this in mind, we investigated the relationship between macular VD parameters and FT in advanced glaucoma patients. In the process, we also determined the associations of macular structural parameters and central VF sensitivity (e.g., central 5° and 10°) with FT, as reference standards.

## Methods

This retrospective, cross-sectional study was approved by the institutional review board of the Asan Medical Center and adhered to the Declaration of Helsinki tenets. The need to obtain informed patient consent was waived by the board due to the retrospective study design. Medical records were obtained from patients who visited the glaucoma clinic of the Asan Medical Center from January 2021 to April 2022.

### Subjects

The initial comprehensive ophthalmic examination of the study subjects comprised a medical history review, best-corrected visual acuity (BCVA) measurement, slit-lamp biomicroscopy, gonioscopy, central corneal thickness (CCT) measurement with ultrasound pachymetry (DGH-550; DGH Technology INC, Exton, PA), Goldmann applanation tonometry (Haag-Streit, Koeniz, Switzerland) intraocular pressure (IOP) measurement, optic disc stereo-photography, red-free RNFL photography, Humphrey field analyzer (HFA) Swedish Interactive Threshold Algorithm (SITA)-Standard 24-2 VF testing (Carl Zeiss Meditec, Dublin, CA), including FT measurement, circumpapillary RNFL (cpRNFL) and macular ganglion cell-inner plexiform layer (mGCIPL) thickness measurement with Cirrus HD spectral-domain OCT (SD-OCT, Carl Zeiss Meditec, Dublin, CA), and OCT-A (AngioVue; Optovue Inc, Fremont, CA). Refractive error was measured with an automated kerato-refractometer (KR-7100, Topcon, Tokyo, Japan).

Open-angle glaucoma (OAG) eyes and healthy control eyes were analyzed consecutively. Inclusion criteria overall were as follows: age > 18 years, spherical equivalent (SE) refractive error within − 6 D to + 3 D, cylinder refraction within − 3 D to + 3 D, normal anterior chamber, and open-angle on slit-lamp and gonioscopic examinations. In the present study, OAG eyes with a VF mean deviation (MD) < − 15 dB at initial presentation were selected to assess the relationship of macular structure, function, and VD measures with FT in advanced stage OAG patients for the following two reasons^19–21^. First, from a structural standpoint, when VF MD was worse than − 14 dB in advanced stage glaucoma, SD-OCT thickness parameters, such as cpRNFL or mGCIPL thickness, approach the measurement floor after which no further structural changes can be detected in SD-OCT measurement^5^. Second, as there are no universally-accepted criteria for the classification of advanced glaucoma, MD < − 15 dB criterion was selected in the present study based on the same criterion used by published studies^21–24^. If both eyes of OAG patients met the inclusion criteria, the eye with worse MD in the VF 24-2 test was analyzed.

OAG was defined as the presence of characteristic glaucomatous optic nerve head (ONH) damage with corresponding typical glaucomatous VF defects. Glaucomatous ONH damage included generalized and/or focal neuroretinal rim thinning, neural rim notching, disc hemorrhage or an RNFL defect, while glaucomatous VF defects were confirmed if a VF met two of the following three criteria:^19^ (1) A cluster of three points with *P* < 0.05 in the pattern deviation map in at least one hemifield (superior/inferior) and at least one point with *P* < 0.01. (2) Glaucoma hemifield test (GHT) result outside normal limits. (3) Pattern standard deviation (PSD) with *P* < 0.05. In eyes with glaucomatous VF defects, the first VF perimetric result was excluded from analysis to preclude a learning effect. The second VF examination was obtained within 2–4 weeks after the first perimetry and was used for analysis.

The healthy control group consisted of the right eyes of subjects from the general eye clinic. They were required to have an IOP < 21 mmHg, with no history of elevated IOP; a normal-appearing ONH, anterior chamber angles, and posterior segment; and normal VF test results (i.e., a PSD within 95% confidence limits and GHT result within normal limits).

Exclusion criteria were a history of uveitis, ocular trauma, and/or intraocular surgery, including cataract or glaucoma surgery, and any macular, retinal, or neurological diseases other than glaucoma that may affect the ONH, macula, or VF examination and BCVA assessment. Eyes with visually significant cataract (based on Lens Opacity Classification System III > C2, N2, or P2) that may induce generalized VF sensitivity depression, including FT measurement, were also excluded^25^. Eyes with a severely elongated globe (axial length > 26 mm) were also excluded to avoid image artifacts associated with high myopia on OCT/OCT-A results.

### cpRNFL and mGCIPL Imaging with OCT

SD-OCT images were acquired using Cirrus HD SD-OCT (version 10.0) software. The optic disc cube scan generated a 6 × 6 × 2 mm^3^ cpRNFL thickness (cpRNFLT) map. Average cpRNFLT was measured within a 3.46-mm diameter circle, and each of four quadrants and 12 clock-hour maps provided sectoral measurements of the cpRNFLT. The average of 5 clock-hours, except the 3 and 9 o’clock sectors, were used to estimate the superior and inferior hemisphere (SH and IH) cpRNFLT value in our analysis, as described previously^12^.

The macular cube 512 × 128 scan mode, which covers 6 × 6-mm^2^ area, generated the mGCIPL thickness (mGCIPLT) map of the annulus region, centered on the fovea. The average, minimum, and six wedge-shaped sectoral (superotemporal, superior, superonasal, inferonasal, inferior, inferotemporal) mGCIPLTs were acquired within the elliptical annulus area, excluding the central foveal region. SH and IH measurements of mGCIPLT were estimated as an average of each three corresponding superior and inferior sectoral values^12^. All cpRNFL and macular OCT scans were reviewed for image quality. Only high-quality cpRNFL and macular scans were included: these images exhibited a centered optic disc, were well-focused, without eye movement, without RNFL or blood vessel discontinuity, misalignment, involuntary saccade or blinking artifacts, or segmentation failure, and had signal strength > 6.

### Macular VD measurement with OCT-A imaging

A macular area OCT-A scan was obtained from each patient using an AngioVue OCT-A system. All OCT-A parameters were obtained using the same version of AngioVue software (version 2018.1.0.43) for consistency. VD was defined as the percentage of area occupied by blood vessels demonstrating flow on OCT-A images. The scan area was 6 × 6 mm centered on the fovea and was divided into the superficial layer (internal limiting membrane [ILM] to 10 μm above the internal plexiform layer [IPL]) and deep layer (10 μm above the IPL to 10 μm below the outer plexiform layer [OPL]). Macular OCT-A imaging of each layer provided the macular whole image, foveal, parafoveal, and perifoveal VD parameters. Macular whole image VD measurements were obtained from images of 6 × 6-mm scans that were centered on the fovea. The foveal VD was measured within a 1-mm diameter circle around the fovea. The parafoveal VD (centered on the fovea, from 1 to 3-mm diameter) and perifoveal VD (centered on the fovea, from 3 to 6-mm diameter) were also measured. Besides the global measurement, SH and IH VD values were separately calculated in the macular whole image, parafovea, and perifovea area. Poor quality OCT-A images were excluded based on the following characteristics: signal strength index < 7; poor clarity due to media opacities; motion artifacts, visualized as an irregular vessel pattern or disc boundary; localized weak signal intensities due to vitreous floater or posterior vitreous detachment; images with a fixation error; or segmentation failure^5,6^.

### Central VF mean sensitivity assessment

The VF 24-2 test was performed using an HFA 750 (Carl Zeiss Meditec) using the SITA-Standard program. The age-adjusted correction was placed in the lens holder for each eye and the FT was tested first, followed by completion of the remaining VF 24-2 test. To assess central VF MS, central 10° and 5° VF MS were obtained as the average values of the differential light sensitivity obtained at the 12 central points and four innermost central points, respectively. The average mean threshold was calculated by converting decibel (dB) values to a 1/L scale [1/Lambert, dB = 10 × log(1/Lambert)] at each locus and averaging these values^12^. We also extracted other VF parameters including the visual field index (VFI), MD, and PSD for the analysis. Only reliable VF-test results (false-positives: < 15%, false-negatives: < 15%, fixation loss: < 20%) were included in the analysis.

### Statistical analyses

All statistical analyses were performed using SPSS (version 26.0, SPSS Inc. Chicago, IL), and R (version 4.0.3, R Foundation for Statistical Computing, Boston, MA) software. In the current study, a reduced FT value associated with advanced glaucoma was regarded as ≤ 31 dB because, first, reduced BCVA associated with advanced glaucoma was found to be a Snellen BCVA of 20/40 or less, which corresponds to a FT value of 31 dB^10^. Second, the median FT value in our advanced glaucoma cohort was 31 dB. Therefore, two subgroups of advanced glaucoma patients based on FT value of 31 (FT ≤ 31 dB vs. FT > 31 dB) and healthy controls were compared using one-way analysis of variance (ANOVA) with the Bonferroni post-hoc test or Kruskal–Wallis analysis with Dunnett post-hoc test for multiple comparisons, depending on the normality of data. The normality of distribution was assessed using a Kolmogorov–Smirnov test. Categorical variables were compared between the groups using Pearson’s chi-square test. Pearson’s correlation and partial correlations, adjusting for age, were used to evaluate correlations of various structural, functional, and VD parameters with FT values in advanced OAG patients, since FT, structure, function, and VD parameters correlate significantly with age^12^. A correlation coefficient value < 0.19 was regarded as very weak, 0.2–0.39 as weak, 0.4–0.59 as moderate, 0.6–0.79 as strong, and 0.8–1.0 as very strong correlation^12^. Receiver operating characteristic (ROC) curve analysis was conducted in advanced OAG eyes to assess the usefulness of different global thickness and VD parameters to detect reduced FT and BCVA values. Areas under the ROC curves (AUCs) of various parameters were compared using the DeLong test. Finally, univariate and multivariate regression analyses were performed to evaluate the association of various covariates with FT. To avoid multicollinearity among the parameters within the same anatomic structures, two different models (i.e., global vs. regional: SH and IH) were separately constructed^26^. In multivariate analysis, variables that were significant at *P* < 0.1 in univariate analyses were included. *P* values < 0.05 were considered to be statistically significant.

## Results

### Comparison of demographics and clinical characteristics

Eighty-six eyes from 86 patients with advanced OAG and 68 eyes from 68 healthy subjects were included in this study. Advanced OAG patients consisted of 50 eyes from 50 patients (FT ≤ 31 dB) and 36 eyes from 36 patients (FT > 31 dB). Table 1 shows various demographic and clinical characteristics of the 3 study groups. Patients in the advanced glaucoma group were older (*P* = 0.020), higher baseline IOP (*P* = 0.011), and had significantly worse BCVA (*P* < 0.001) than subjects in the healthy control group. Sex and CCT were not significantly different among the three groups (*P* = 0.986 and *P* = 0.814, respectively). VF parameters, including FT, VFI, MD, central 10° VF MS, and central 5° VF MS, differed significantly among the three groups (all *P* < 0.001). Similarly, structure, function, superficial and deep VD measures demonstrated statistically significant differences among the three groups (all *P* < 0.001).

**Table 1:** Comparison of demographic and clinical characteristics between the advanced glaucoma patients and healthy controls.

Parameters	Advanced glaucoma groups (n = 86)		(C) Healthy control	*P* value	Post-Hoc significance
	(A) FT ≤ 31 dB	(B) FT > 31 dB			
Patients (n)	50	36	68		
Age (y)**	64.0 (58.0, 73.0)	62.0 (45.5, 68.0)	54.5(50.0, 64.0)	0.020	A = B > C
Sex (Male/Female)***	30/20	21/15	40/28	0.986	
SE (Diopter)**	− 2.1 (− 5.2, − 0.2)	− 0.8 (− 2.9, 0.2)	− 0.2 (− 1.2, 0.2)	< 0.001	A < B < C
IOP (mmHg)**	15.0 (12.5, 17.0)	14.0 (12.0, 17.2)	13.0 (12.0, 15.0)	0.011	A = B > C
CCT (μm)*	539.1 ± 41.9	533.1 ± 45.5	537.0 ± 41.4	0.814	
LogMAR VA**	0.3 (0.2, 0.7)	0.1 (0.1, 0.1)	0.0 (0.0, 0.0)	< 0.001	A > B > C
Foveal threshold (dB)*	26.6 ± 4.47	34.4 ± 2.13	36.4 ± 1.76	< 0.001	A < B < C
VF index (%)*	25.4 ± 14.6	37.0 ± 13.2	99.2 ± 0.87	< 0.001	A < B < C
MD (dB)*	-24.7 ± 4.04	-22.0 ± 3.92	0.06 ± 1.10	< 0.001	A < B < C
PSD (dB)*	9.54 ± 2.40	11.8 ± 2.36	1.76 ± 1.54	< 0.001	A < C < B
Central 10° VF MS (dB)*	8.21 ± 4.23	12.4 ± 4.02	32.2 ± 1.40	< 0.001	A < B < C
Central 5° VF MS (dB)*	11.1 ± 5.34	17.6 ± 5.49	33.0 ± 1.36	< 0.001	A < B < C
Average cpRNFLT (μm)*	61.1 ± 7.68	61.2 ± 8.34	96.5 ± 7.52	< 0.001	A = B < C
Average mGCIPLT (μm)*	54.8 ± 6.92	57.6 ± 6.53	81.9 ± 3.53	< 0.001	A = B < C
**OCT-A Superficial macular VD**					
Macular whole image (%)*	31.7 ± 4.07	34.7 ± 3.50	48.6 ± 4.36	< 0.001	A < B < C
Foveal (%)*	12.2 ± 7.31	15.9 ± 7.48	18.2 ± 7.05	< 0.001	A = B < C
Global parafoveal (%)*	35.2 ± 4.89	39.7 ± 5.05	50.9 ± 5.28	< 0.001	A < B < C
Global perifoveal (%)*	31.8 ± 4.23	34.5 ± 3.76	49.9 ± 4.48	< 0.001	A < B < C
**OCT-A Deep macular VD**					
Macular whole image (%)*	36.4 ± 6.38	40.5 ± 6.76	50.5 ± 5.62	< 0.001	A < B < C
Foveal (%)*	24.7 ± 6.98	28.8 ± 7.12	35.0 ± 6.92	< 0.001	A < B < C
Global parafoveal (%)*	42.2 ± 6.59	47.7 ± 6.04	55.2 ± 4.12	< 0.001	A < B < C
Global perifoveal (%)*	36.5 ± 6.73	40.6 ± 7.42	51.5 ± 6.27	< 0.001	A < B < C

Data with normal distribution were shown as mean ± standard deviation, otherwise data with non-normal distribution were shown as median (25th, 75th percentile).

FT, foveal threshold; n, number; SE, spherical equivalent; IOP, intraocular pressure; CCT, central corneal thickness; VA, visual acuity; VF, visual field; MD, mean deviation; PSD, pattern standard deviation; MS, mean sensitivity; cpRNFLT, circumpapillary retinal nerve fiber layer thickness; mGCIPLT, macular ganglion cell-inner plexiform layer thickness; OCT-A, optical coherence tomography angiography; VD, vessel density.

*One-way ANOVA was used for normally distributed data with Bonferroni post-hoc test for multiple comparisons, **Kruskal–Wallis analysis was used for non-normally distributed data with Dunnett post-hoc test for multiple comparisons, ***Pearson’s chi-square test was used for categorial variables.

### Correlations of structure, function, and VD parameters with FT

Various structure, function, and VD measurements showed significant correlations with FT, after adjusting for age (Table 2). Among structural parameters, the average mGCIPLT and SH mGCIPLT were significantly correlated with FT, after adjusting for age. All of the central 10° and 5° VF MS parameters, regardless of location (i.e., SH, IH, or global), were significantly correlated with FT. Among the functional parameters, LogMAR BCVA (r = -0.73, *P* < 0.001) and central VF 5° MS (r = 0.61, *P* < 0.001) showed the highest correlations with FT after age adjustment. All macular VD parameters of the superficial and deep layers showed significant correlations with FT, including global and hemispheric measurements of the macular whole image, parafoveal, and perifoveal VD. Among macular VD parameters, superficial global and SH parafoveal VD showed the highest correlations with FT (superficial global parafoveal VD: r = 0.47, *P* < 0.001, superficial SH parafoveal VD: r = 0.47, *P* < 0.001), followed by deep global parafoveal VD (r = 0.45, *P* < 0.001). With respect to various anatomical locations, parafoveal VD parameters in both the superficial and deep macula showed relatively stronger correlations with FT than did the macular whole image, fovea, or perifoveal VD parameters.

**Table 2:** Analyses of the correlation of structure, function, and vessel density parameters with the foveal threshold in advanced glaucoma patients.

Parameters	Advanced glaucoma group (n = 86)	
	Pearson correlation coefficient (*P* value)	Partial correlation coefficient with age adjustment (*P* value)
LogMAR visual acuity	− 0.72 (< 0.001)	− 0.73 (< 0.001)
**Visual field MS**		
Central 10° VF Ms	0.49 (< 0.001)	0.48 (< 0.001)
Central 10° SH VF MS	0.24 (0.024)	0.24 (0.028)
Central 10° IH VF MS	0.42 (< 0.001)	0.40 (< 0.001)
Central 5° VF MS	0.62 (< 0.001)	0.61 (< 0.001)
Central 5° SH VF MS	0.36 (0.001)	0.34 (0.001)
Central 5° IH VF MS	0.52 (< 0.001)	0.52 (< 0.001)
**OCT parameters**		
Average cpRNFLT	− 0.02 (0.829)	− 0.01 (0.934)
cpRNFLT SH	0.01 (0.938)	0.02 (0.869)
cpRNFLT IH	− 0.05 (0.637)	− 0.03 (0.789)
Average mGCIPLT	0.18 (0.104)	0.23 (0.032)
mGCIPLT SH	0.19 (0.079)	0.25 (0.021)
mGCIPLT IH	0.07 (0.534)	0.12 (0.258)
**OCT-A Vessel density**		
Superficial		
Macular whole image	0.44 (< 0.001)	0.44 (< 0.001)
Macular SH	0.42 (< 0.001)	0.42 (< 0.001)
Macular IH	0.34 (0.001)	0.35 (0.001)
Foveal	0.25 (0.023)	0.25 (0.021)
Global parafoveal	0.47 (< 0.001)	0.47 (< 0.001)
Parafoveal SH	0.48 (< 0.001)	0.47 (< 0.001)
Parafoveal IH	0.37 (< 0.001)	0.37 (< 0.001)
Global perifoveal	0.41 (< 0.001)	0.41 (< 0.001)
Perifoveal SH	0.38 (< 0.001)	0.37 (< 0.001)
Perifoveal IH	0.40 (< 0.001)	0.40 (< 0.001)
Deep		
Macular whole image	0.38 (< 0.001)	0.36 (0.001)
Macular SH	0.37 (< 0.001)	0.35 (0.001)
Macular IH	0.36 (0.001)	0.35 (0.001)
Foveal	0.41 (< 0.001)	0.40 (< 0.001)
Global parafoveal	0.46 (< 0.001)	0.45 (< 0.001)
Parafoveal SH	0.44 (< 0.001)	0.43 (< 0.001)
Parafoveal IH	0.44 (< 0.001)	0.43 (< 0.001)
Global perifoveal	0.35 (0.001)	0.34 (0.002)
Perifoveal SH	0.36 (0.001)	0.34 (0.002)
Perifoveal IH	0.32 (0.002)	0.31 (0.004)

VF MS = visual field mean sensitivity, OCT = optical coherence tomography, cpRNFLT, circumpapillary retinal nerve fiber layer thickness; mGCIPLT, macular ganglion cell-inner plexiform layer thickness; SH, superior hemisphere; IH, inferior hemisphere; OCT-A, optical coherence tomography angiography.

### AUC analysis of the discrimination of decreased FT and BCVA using structure and VD parameters

Table 3 shows that all of the superficial and deep layer macular VD parameters had significant AUC values (all *P* < 0.05) for detecting reduced FT and BCVA in our advanced glaucoma group, while none of the structural parameters revealed significant AUC values. Among various VD parameters, superficial and deep global parafoveal VD showed the highest AUC values, which fit well with the results of the correlation analyses in our study. According to the DeLong test (*P* value of Table 3A and B, respectively), the AUC of deep global parafoveal VD was significantly greater than that of the average cpRNFLT (*P* = 0.001, *P* = 0.008), deep macular whole image VD (*P* = 0.018, *P* = 0.015), and deep global perifoveal VD (*P* = 0.019, *P* = 0.014), while the AUC of the superficial global parafoveal VD was significantly greater than that of the average cpRNFLT (*P* = 0.005, *P* = 0.019). The cutoff values, sensitivity, and specificity of different OCT/OCT-A parameters of decreased FT and BCVA are presented in Table 3.

**Table 3:** The area under the receiver operating characteristic (ROC) curve analysis for optical coherence tomography (OCT) and OCT-angiography (OCT-A) parameters as predictors of decreased central visual function in advanced glaucoma patients.

(A) ROC curve analysis for detecting decreased foveal threshold (FT ≤ 31 dB)						
Parameters	Advanced glaucoma group (n = 86)					
	AUC	95% CI	*P* value	Cutoff value	Sensitivity	Specificity
**OCT parameters**						
Average cpRNFLT	0.501	0.392–0.611	0.983	47.0	91.67	0.00
Average mGCIPLT	0.617	0.506–0.720	0.059	56.0	52.78	72.00
**OCT-A Vessel density**						
Superficial						
Macular whole image	0.716	0.609–0.808	**0.001**	32.5	80.56	62.00
Foveal	0.679	0.570–0.776	**0.002**	11.6	75.00	68.00
Global parafoveal	0.728	0.621–0.818	** < 0.001**	36.4	75.00	62.00
Global perifoveal	0.698	0.589–0.792	**0.001**	32.1	80.56	58.00
Deep						
Macular whole image	0.672	0.554–0.789	**0.005**	40.4	58.33	78.00
Foveal	0.664	0.554–0.763	**0.006**	27.3	66.67	68.00
Global parafoveal	0.738	0.632–0.827	** < 0.001**	47.8	55.56	88.00
Global perifoveal	0.667	0.557–0.765	**0.006**	41.4	58.33	80.00

(B) ROC curve analysis for detecting decreased Snellen best-corrected visual acuity (Snellen BCVA ≤ 20/40)						
Parameters	Advanced glaucoma group (n = 86)					
	AUC	95% CI	*P* value	Cutoff value	Sensitivity	Specificity
**OCT parameters**						
Average cpRNFLT	0.524	0.401–0.648	0.699	57.0	74.36	38.30
Average mGCIPLT	0.618	0.499–0.737	0.060	56.0	51.28	72.34
**OCT-A Vessel density**						
Superficial						
Macular whole image	0.699	0.589–0.810	**0.002**	31.9	79.49	59.57
Foveal	0.696	0.584–0.808	**0.002**	11.6	74.36	70.21
Global parafoveal	0.720	0.613–0.826	** < 0.001**	36.2	74.36	61.70
Global perifoveal	0.677	0.564–0.791	**0.005**	30.3	89.74	46.81
Deep						
Macular whole image	0.652	0.536–0.769	**0.015**	40.4	53.85	76.60
Foveal	0.694	0.581–0.806	**0.002**	26.2	69.23	68.09
Global parafoveal	0.720	0.611–0.828	** < 0.001**	47.8	51.28	87.23
Global perifoveal	0.646	0.528–0.765	**0.020**	41.4	53.85	78.72

Statistically significant values are in bold.

OCT, optical coherence tomography; OCT-A, optical coherence tomography angiography; FT, foveal threshold; AUC, area under the curve; CI, confidence interval; cpRNFLT, circumpapillary retinal nerve fiber layer thickness; mGCIPLT, macular ganglion cell-inner plexiform layer thickness.

### Linear regression analyses between glaucoma parameters and FT

**Figure 1:**
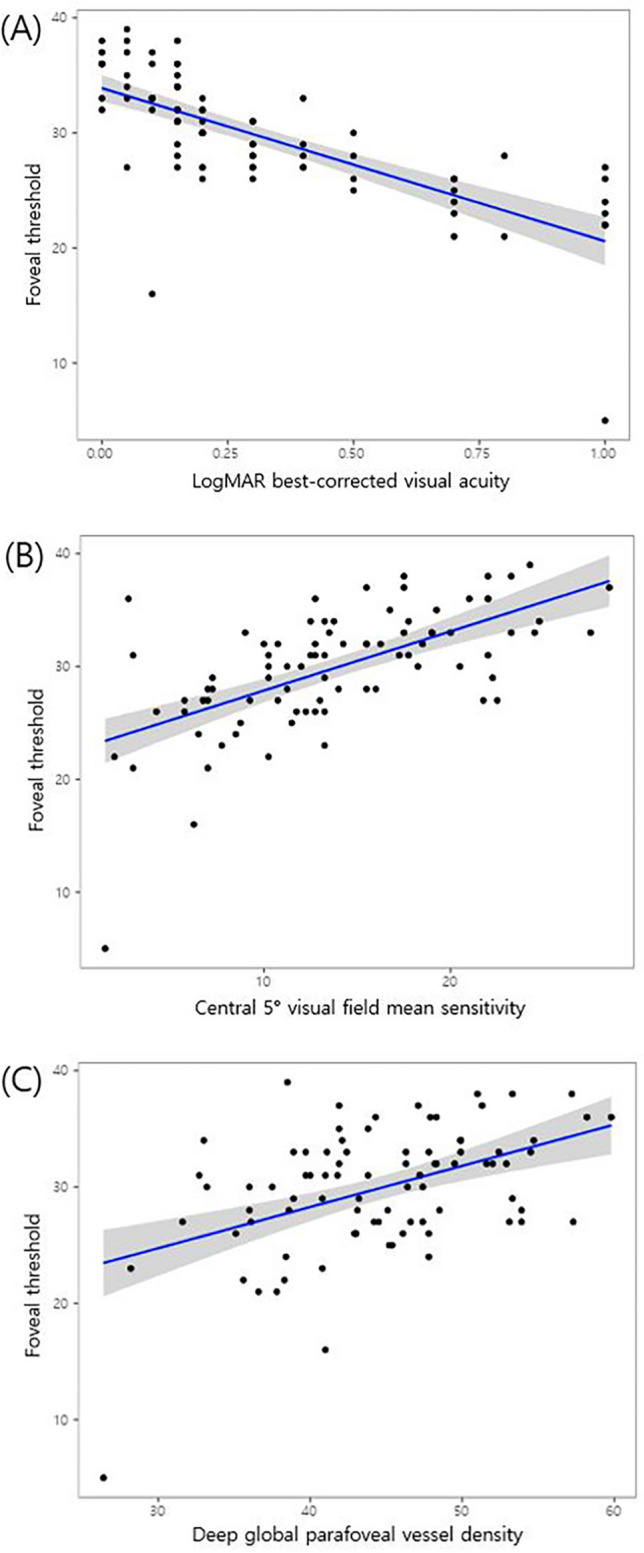
Scatterplot and best fit-line between foveal threshold and various covariates. (**A**) Foveal threshold by LogMAR best-corrected visual acuity (r^2^ = 0.518, *P* < 0.001, linear regression analysis), (**B**) foveal threshold by central 5° visual field mean sensitivity (r^2^ = 0.386, *P* < 0.001, linear regression analysis), and (**C**) foveal threshold by deep global parafoveal vessel density (r^2^ = 0.212, *P* < 0.001, linear regression analysis).

Table 4 illustrates the association of various demographic, structure, function, and macular VD variables with FT. In the univariate analyses, FT was significantly related to all VF indices and OCT-A parameters, while none of the OCT-derived thickness parameters, including mGCIPLT, were associated with FT. In multivariate analyses, LogMAR BCVA (β = − 8.80, *P* < 0.001), central 5° VF MS (β = 0.30, *P* = 0.037), SH central 5° VF MS (β = 0.28, *P* = 0.009), IH central 5° VF MS (β = 0.27, *P* = 0.011), and deep global parafoveal VD (β = 0.37, *P* = 0.037) were significantly associated with FT. The scatterplot and best-fit line between these parameters and FT are visually illustrated in Fig. 1. Among the various macular structure and VD parameters, only the deep parafoveal global VD was found to be significantly associated with FT in our multivariate analyses.

**Table 4:** Linear regression analyses for the association of clinical characteristics and structure, function, and vessel density parameters with foveal threshold in advanced glaucoma patients.

Parameters	Univariate			Multivariate I (LogMAR BCVA, MD, Central 10° VF MS, Central 5° VF MS, macular whole image VD, foveal VD, superficial & deep global parafoveal and perifoveal VD			Multivariate II (SH & IH factors : Central 10° VF MS, Central 5° VF MS, mGCIPLT SH, macular VD, superficial & deep parafoveal and perifoveal VD)		
	β	SE	*P* value	Β	SE	*P* value	β	SE	*P* value
Age	− 0.06	0.04	0.182						
SE	− 0.03	0.16	0.862						
IOP	− 0.13	0.16	0.414						
CCT	− 0.01	0.01	0.534						
LogMAR BCVA	− 13.30	1.40	< 0.001	− **8.80**	**1.74**	** < 0.001**			
**Visual field parameters**									
VFI	0.16	0.03	< 0.001						
MD	0.46	0.13	< 0.001						
PSD	1.01	0.19	< 0.001						
Central 10° VF MS	0.56	0.11	< 0.001						
Central 10° SH VF MS	0.22	0.10	0.024						
Central 10° IH MS	0.30	0.07	< 0.001						
Central 5° VF MS	0.53	0.07	< 0.001	**0.30**	**0.14**	**0.037**			
Central 5° SH VF MS	0.24	0.07	< 0.001				**0.28**	**0.11**	**0.009**
Central 5° IH VF MS	0.29	0.05	< 0.001				**0.27**	**0.10**	**0.011**
**OCT parameters**									
Average cpRNFLT	− 0.02	0.07	0.829						
cpRNFLT SH	0.00	0.06	0.938						
cpRNFLT IH	-0.03	0.07	0.637						
Average mGCIPLT	0.14	0.08	0.104						
mGCIPLT SH	0.16	0.09	0.079						
mGCIPLT IH	0.06	0.09	0.534						
**OCT-A Vessel density**									
Superficial									
Macular whole image	0.57	0.13	< 0.001						
Macular SH	0.49	0.12	< 0.001						
Macular IH	0.42	0.13	0.010						
Foveal	0.17	0.07	0.023						
Global parafoveal	0.46	0.09	< 0.001						
Parafoveal SH	0.42	0.08	< 0.001						
Parafoveal IH	0.36	0.10	< 0.001						
Global perifoveal	0.51	0.12	< 0.001						
Perifoveal SH	0.42	0.11	< 0.001						
Perifoveal IH	0.54	0.13	< 0.001						
Deep									
Macular whole image	0.29	0.08	< 0.001						
Macular SH	0.28	0.08	< 0.001						
Macular IH	0.28	0.08	< 0.001						
Foveal	0.30	0.07	< 0.001						
Global parafoveal	0.35	0.07	< 0.001	**0.37**	**0.18**	**0.037**			
Parafoveal SH	0.33	0.07	< 0.001						
Parafoveal IH	0.32	0.07	< 0.001						
Global perifoveal	0.26	0.07	< 0.001						
Perifoveal SH	0.25	0.07	< 0.001						
Perifoveal IH	0.23	0.07	0.002						

Statistically significant values are in bold.

VF MS, visual field mean sensitivity; β, standardized beta coefficient; SE, spherical equivalent; IOP, intraocular pressure; CCT, central corneal thickness; BCVA, best-corrected visual acuity; VFI, visual field index; MD, mean deviation; PSD, pattern standard deviation; SH, superior hemisphere; IH, inferior hemisphere; OCT, optical coherence tomography; OCT-A, optical coherence tomography angiography; cpRNFLT, circumpapillary retinal nerve fiber layer thickness; mGCIPLT, macular ganglion cell-inner plexiform layer thickness.

### Representative cases

**Figure 2:**
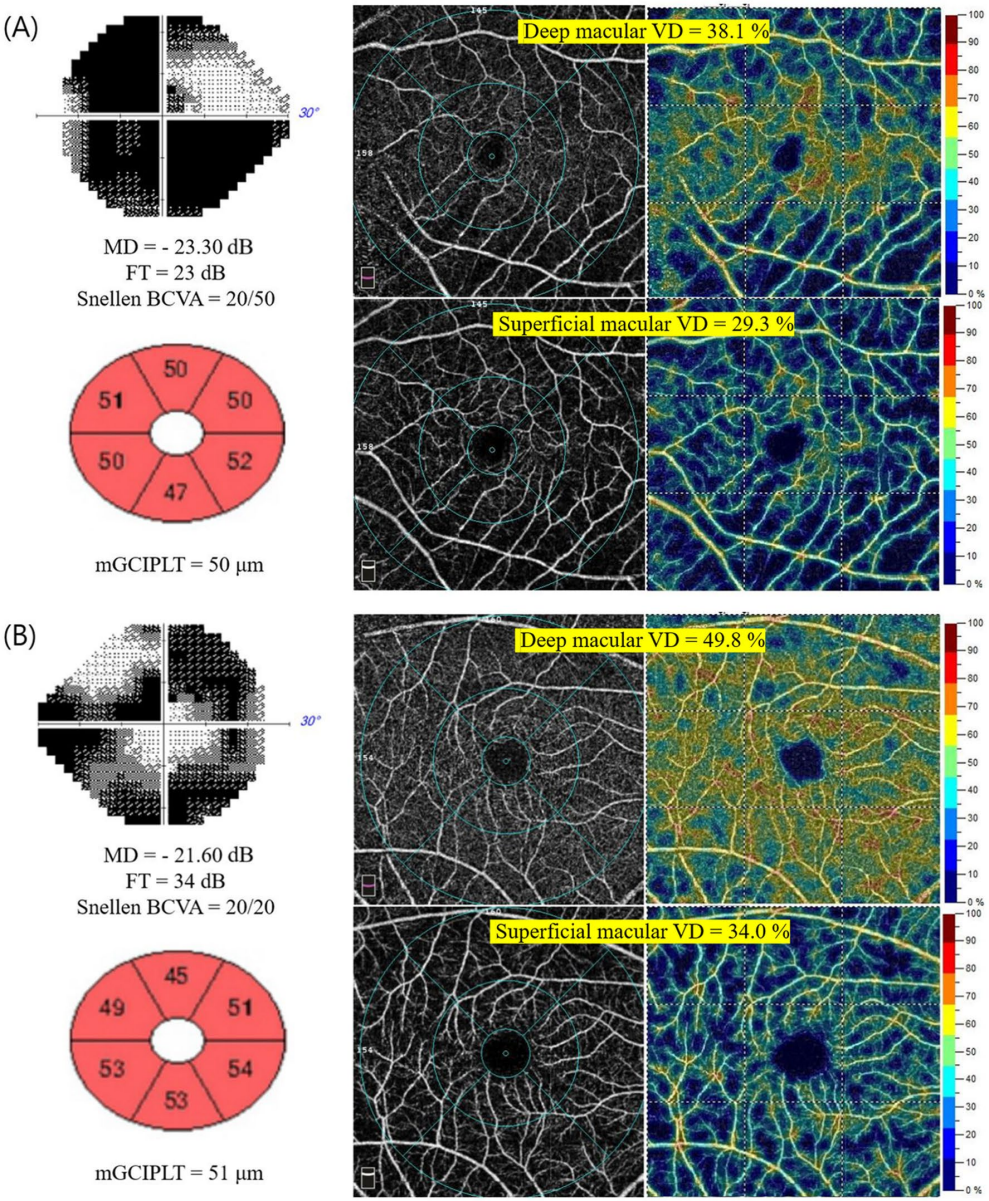
Two representative cases of advanced open-angle glaucoma with similar visual field (VF) mean deviation (MD) and macular ganglion cell-inner plexiform layer thickness (mGCIPLT) and different central visual function (CVF) parameters. (**A**) Advanced OAG case with CVF impairment. Optical coherence tomography angiography (OCT-A) en-face image (deep layer; top middle, superficial layer; bottom middle) and macular vessel density (mVD) map (deep layer; top right, superficial layer; bottom right) reveal that the deep and superficial layer mVDs are 38.1% and 29.3%, respectively. (**B**) Advanced OAG case with CVF preservation. OCT-A en-face image (deep layer; top middle, superficial layer; bottom middle) and mVD map (deep layer; top right, superficial layer; bottom right) demonstrate that the deep and superficial layer mVDs are 49.8% and 34.0%, respectively. Deep layer mVD loss is more pronounced (38.1% vs. 49.8%) in the eye with CVF impairment (FT = 23 dB, **A**) than in the eye with CVF preservation (FT = 34 dB, **B**) despite both cases having similar VF MD (− 23.30 dB vs. − 21.60 dB), mGCIPLT (50 μm vs. 51 μm), and superficial layer mVD (29.3% vs. 34.0%). These findings indicate that deep mVD correlates well with CVF parameter, such as FT, in eyes with advanced glaucoma. VF, visual field; CVF, central visual function; VD, vessel density; OCT-A, optical coherence tomography angiography.

Figure 2 shows representative cases (A, B). Figure 2A demonstrates an advanced OAG case with a VF MD of − 23.30 dB and CVF impairment, represented by decreased FT and Snellen BCVA with 23 dB and 20/50, respectively. The superficial and deep macular VD derived from OCT-A are 29.3% and 38.1%, respectively. The average mGCIPLT measured by SD-OCT is 50 μm, which appears to have reached the measurement floor. Figure 2B depicts an advanced OAG case with VF MD of − 21.60 dB with relatively intact CVF, represented by FT and Snellen BCVA of 34 dB and 20/20, respectively. mGCIPLT shows an average value of 51 μm. The superficial and deep macular VD values are 34.0% and 49.8%, respectively. Of note, deep macular VD loss is more pronounced in the eye with CVF impairment (A) than in the eye with CVF preservation (B) (38.1% vs. 49.8%), despite both cases having similar VF MD, mGCIPLT, and superficial layer mVD (29.3% vs. 34.0%), indicating that deep macular VD associates well with CVF parameters, including FT and BCVA, in advanced glaucomatous eyes.

## Discussion

In this study, we evaluated whether there was a relationship between various macular VD parameters and FT in eyes with advanced glaucoma, as that would allow macular VD parameters to be used as a surrogate marker for estimating CVF and monitoring its change over time in advanced glaucoma cases, as FT is often preserved to the late stage of glaucoma and is closely linked to CVF^26^. We found that all of the macular VD parameters at both superficial and deep layers, as well as VF MS within the central 5° and 10°, showed significant correlations with FT after adjustment for age. Furthermore, multivariate analyses showed that FT was independently associated with deep global parafoveal VD, BCVA, and central 5° VF MS, after controlling for other covariates. No previous study has evaluated the relationship between macular VD parameters of two distinct layers and FT, representing CVF, in advanced glaucoma eyes. Our findings may provide new insight into the clinical utility of macular VD parameters, particularly deep global parafoveal VD, in predicting CVF of eyes with advanced glaucoma.

FT is recognized as an important surrogate measure of CVF. FT is significantly correlated with BCVA, suggesting that FT is a sensitive foveal measure of visual function^10,11^. Weiner et al.^27^ reported a decrease in amplitude on the foveal cone electroretinogram in glaucomatous eyes. Jeong et al.^12^ reported that FT in early-to-moderate stage glaucoma patients was significantly lower than that of healthy eyes, and that BCVA was positively associated with FT. In the current study, it was not surprising that FT was also significantly reduced in advanced glaucoma eyes as compared to that of healthy control eyes (Table 1), which was consistent with the findings of a previous study^12^, except that the present study involved eyes with far greater glaucoma severity.

In the current study, while central 5° and 10° VF MS correlated significantly with FT, central 5° VF MS showed a stronger correlation with FT than did central 10° VF MS (r = 0.61 vs. 0.48, respectively), which may be explained by the anatomical proximity of the central 5° to the fovea^28^. Of note, the correlation coefficient between central 10° VF MS and FT was found higher for eyes with advanced glaucoma in the current study than that of eyes with early-to-moderate glaucoma reported by Jeong et al.^12^ (r = 0.48 vs. 0.361, respectively). One explanation for this finding is the higher frequency of central VF loss involving the central 10° area in eyes with advanced glaucoma (MD < − 15 dB). Since CVF parameters, such as central 10° VF MS and FT, are often affected in late-stage glaucoma with extensive VF loss, it is plausible that the central 10° VF MS showed a higher correlation with FT in eyes with advanced glaucoma than in eyes with early-to-moderate stage glaucoma, which have a relatively intact central 10° VF. Our findings may further support the role of FT as a surrogate marker for CVF in advanced glaucoma.

Of note, while the average and SH mGCIPLT correlated significantly with FT, there was no significant correlation between cpRNFLT and FT in the present study (Table 2). There are a few possible explanations for these findings. Since the measurement locations of cpRNFLT and mGCIPLT are the circumpapillary and macula areas, respectively, mGCIPLT is more likely to be correlated with FT than cpRNFLT, considering the anatomical proximity of the macula to the fovea. In addition, the floor effect of the structural parameter (i.e., cpRNFLT) in advanced glaucoma may contribute to the lack of association between cpRNFLT and FT. The measurement floor is lower in the order of macular VD, mGCIPLT, and cpRNFLT, based on published studies^4,5,29–31^, in which cpRNFLT is known to reach the measurement floor approximately at an MD value of − 10 to − 15 dB, while mGCIPLT reaches the measurement floor at a lower level. In this study, since cpRNFL had already reached the measurement floor in our advanced glaucoma patients, there were no significant correlations between cpRNFL parameters and FT (Table 2)^29–32^. Our findings were consistent with those of previous studies, showing that macular structural parameters, such as mGCIPLT, showed a greater degree of structure–function relationship with CVF than cpRNFLT in advanced-stage glaucoma eyes^14,33–35^.

In recent years, OCT-A imaging has increasingly been utilized for the diagnosis and monitoring of glaucoma. One possible advantage of OCT-A imaging in glaucoma is that OCT-A-derived VD parameters have a lower measurement floor than conventional structural OCT parameters, including mGCIPLT^5,36,37^. Therefore, macular VD parameters may have potential for application in monitoring disease progression in severe cases of glaucoma, in which glaucoma patients have reached the measurement floor with OCT thickness parameters (i.e., cpRNFLT or mGCIPLT). In the current study, although the degree of correlation was moderate, both superficial and deep macular VD (i.e., global parafoveal VD; r = 0.47, 0.45, respectively) showed significant correlations with FT in our advanced glaucoma patients (Table 2). Of interest, the degree of correlation between FT and global parafoveal VD was higher than that between FT and perifoveal VD, regardless of the layer (i.e., superficial vs. deep). This is thought to be due to the anatomical proximity of parafoveal region to the foveal center.

In the present study, we also calculated AUC values for the discrimination of decreased CVF, defined as a FT ≤ 31 dB or Snellen BCVA ≤ 20/40, for global OCT/OCT-A parameters. All of the superficial and deep macular VD parameters, including macular whole image and global parafoveal and perifoveal VD parameters, but none of the OCT thickness parameters (*P* > 0.05, Table 4), showed significant AUC values for discriminating reduced FT and BCVA (all *P* < 0.05). These findings agreed with published studies investigating the relationship between VF MS and OCT-A^4,5,29–31^, which have demonstrated that VD had a lower measurement floor than did OCT thickness parameters and was a potential marker for disease monitoring in the advanced stage of glaucoma.

OCT-A provides an estimation of macular VD at each superficial and deep layer, in which superficial VD is found to be reduced in glaucoma^38–40^. However, while the superficial layer macular VD decreases and is correlated with ganglion cell loss and VF defects in early-to-moderate glaucoma^14,33–35^, it remains unclear whether deep macular VD is affected in various glaucoma stages. With disease progression, deep macular VD may begin to show microvascular compromise, since the deep micro-vessels have free anastomoses with the superficial vessels^41–44^. Hsia et al.^6^ reported that deep macular VD was reduced in severe glaucoma and showed a higher correlation with VA than did any other structural parameters. Yoshikawa et al.^45^ also reported that quantitative reduction and vertical asymmetry of deep macular VD is related to glaucomatous central visual loss. According to our linear regression analyses, while both the superficial and deep macular VD parameters showed significant associations with FT in the univariate analyses, our multivariate analyses revealed that only deep global parafoveal VD was a significant predictor of FT value, after controlling for other macular VD parameters, in our series of eyes with advanced glaucoma (Table 3). Our study was in line with Hsia et al.’s report^6^ that deep macular VD may be a promising marker for monitoring disease progression in severe glaucoma, even though the functional outcomes used in the two studies differed (VA vs. FT).

This study had several limitations. In the present study, the threshold values of the central 5° and 10° of the VF 24-2 test were used to represent one measure of CVF. However, it has been shown that the VF 10-2 or VF 24-2C programs may provide more detailed information on the detection of central VF defects and have a better structure–function concordance than VF 24-2^46–48^. In particular, since the VF 10-2 test is more sensitive for the evaluation of disease progression in advanced glaucoma, analysis using VF 10-2 will be needed for better elucidation of the relationship between VF 10-2 VF and FT in future studies. Another limitation is the definition of advanced glaucoma (MD < − 15 dB) utilized in this study, which is relatively arbitrary. Nonetheless, we selected this criterion based on the information related to the measurement floor of SD-OCT thickness parameters^5^ and the same criterion used by previous reports^22–24^ as the purpose of our study was to find a surrogate marker for CVF beyond the thickness parameters provided by SD-OCT. However, the definition of advanced glaucoma should be considered more comprehensively in the context of the location of VF defects or the use of a specific grading system. Despite inclusion of only reliable VF, OCT, and OCT-A results for analysis in this study, the test–retest variability may have affected the outcome analyses as a confounding factor in this cross-sectional study, particularly in eyes with advanced glaucoma. Since increased test–retest variability of the VF 24-2 test is known to occur in advanced glaucoma^49^, analysis of longitudinal data with a large test dataset may provide more accurate information regarding the associations of various structure, function, VD parameters with FT. In addition, as macular VD derived from OCT-A imaging is known to be affected by systemic and ocular factors, such as diabetes, hypertension, and use of systemic and ocular anti-hypertensive medications^50–52^, our study outcomes should be interpreted in the context of these confounding effects, since our study subjects were not screened for systemic disease. Finally, lens opacity can affect the measurement of OCT/OCT-A as well as the sensitivity threshold of FT and the VF 24-2 test^53^. However, we attempted to minimize the impact of media opacity, such as cataract, on our study outcomes by strictly applying Lens Opacities Classification System criteria during our enrollment of both the study and the control group^25^.

In conclusion, macular structure, function, and VD parameters, including mGCIPLT, central 5° and 10° VF MS, and superficial and deep macular VD parameters, were significantly correlated with FT in eyes with advanced glaucoma. In our multivariate analyses, however, deep global parafoveal VD along with BCVA and central 5° VF MS were independent predictors of FT, after controlling for other covariates. Our findings suggest that deep-layer parafoveal VD may have a potential role as a surrogate marker for monitoring CVF in advanced glaucoma.

## Data Availability

The datasets generated and analyzed during the current study are available from the corresponding author on reasonable request.

## References

[1] Weinreb RN, Aung T, Medeiros FA (2014). The pathophysiology and treatment of glaucoma: A review. JAMA.

[2] Malik R, Swanson WH, Garway-Heath DF (2012). Structure-function relationship' in glaucoma: Past thinking and current concepts. Clin. Exp. Ophthalmol..

[3] Ajtony C, Balla Z, Somoskeoy S, Kovacs B (2007). Relationship between visual field sensitivity and retinal nerve fiber layer thickness as measured by optical coherence tomography. Invest. Ophthalmol. Vis. Sci..

[4] Bowd C, Zangwill LM, Weinreb RN, Medeiros FA, Belghith A (2017). Estimating optical coherence tomography structural measurement floors to improve detection of progression in advanced glaucoma. Am. J. Ophthalmol..

[5] Moghimi S, Bowd C, Zangwill LM (2019). Measurement floors and dynamic ranges of OCT and OCT angiography in glaucoma. Ophthalmology.

[6] Hsia Y, Wang T-H, Huang J-Y, Su C-C (2022). Relationship between macular microvasculature and visual acuity in advanced and severe glaucoma. Am. J. Ophthalmol..

[7] Rao HL, Begum VU, Khadka D, Mandal AK, Senthil S, Garudadri CS (2015). Comparing glaucoma progression on 24–2 and 10–2 visual field examinations. PLoS ONE.

[8] Crabb, D. P. *et al.* Frequency of visual field testing when monitoring patients newly diagnosed with glaucoma: mixed methods and modelling. *Health Serv. Deliv. Res.* **2**(27) (2014). 25642569

[9] Hashimoto Y, Kiwaki T, Sugiura H (2021). Predicting 10–2 visual field from optical coherence tomography in glaucoma using deep learning corrected with 24–2/30-2 visual field. Transl. Vis. Sci. Technol..

[10] Flaxel CJ, Samples JR, Dustin L (2007). Relationship between foveal threshold and visual acuity using the Humphrey visual field analyzer. Am. J. Ophthalmol..

[11] Ozeki N, Yuki K, Shiba D, Tsubota K (2017). Evaluation of functional visual acuity in glaucoma patients. J. Glaucoma.

[12] Jeong D, Won HJ, Jo YH, Song MK, Shin JW, Kook MS (2020). Relationship between foveal threshold and macular Structure/Function/Vessel density in glaucoma. J. Glaucoma.

[13] Anderson DR, Patella VM (1999). Automated Static Perimetry.

[14] Shin JW, Lee J, Kwon J (2019). Relationship between macular vessel density and central visual field sensitivity at different glaucoma stages. Br. J. Ophthalmol..

[15] Song MK, Shin JW, Jo Y, Won HJ, Kook MS (2021). Relationship between peripapillary vessel density and visual field in glaucoma: A broken-stick model. Br. J. Ophthalmol..

[16] Wu J-H, Moghimi S, Nishida T, Mahmoudinezhad G, Zangwill LM, Weinreb RN (2022). Association of macular vessel density and ganglion cell complex thickness with central visual field progression in glaucoma. Br. J. Ophthalmol..

[17] Lin F, Li F, Gao K (2021). Longitudinal changes in macular optical coherence tomography angiography metrics in primary open-angle glaucoma with high myopia: A prospective study. Invest. Ophthalmol. Vis. Sci..

[18] Rao HL, Pradhan ZS, Weinreb RN (2017). Relationship of optic nerve structure and function to peripapillary vessel density measurements of optical coherence tomography angiography in glaucoma. J. Glaucoma.

[19] Hodapp E, Parrish RK, Anderson DR (1993). Clinical Decisions in Glaucoma.

[20] Forchheimer I, De Moraes C, Teng C (2011). Baseline mean deviation and rates of visual field change in treated glaucoma patients. Eye.

[21] Anderson DR, Patella VM (1992). Automated Static Perimetry.

[22] Jammal AA, Ferreira BG, Zangalli CS (2020). Evaluation of contrast sensitivity in patients with advanced glaucoma: Comparison of two tests. Br. J. Ophthalmol..

[23] Araie M, Hori J, Koseki N (1995). Comparison of visual field defects between normal-tension and primary open-angle glaucoma in the late stage of the disease. Graefes Arch. Clin. Exp. Ophthalmol..

[24] Suzumura H, Yoshikawa K, Kimura T, Nanno M, Tsumura T (2022). Cluster formation for analyses of glaucomatous visual field defects in central 10–2 visual field in normal tension glaucoma eyes. Clin. Ophthalmol. (Auckland, NZ)..

[25] Chylack LT, Wolfe JK, Singer DM (1993). The lens opacities classification system III. Arch. Ophthalmol..

[26] Raza AS, Cho J, de Moraes CG (2011). Retinal ganglion cell layer thickness and local visual field sensitivity in glaucoma. Arch. Ophthalmol..

[27] Weiner A, Ripkin DJ, Patel S, Kaufman SR, Kohn HD, Weidenthal DT (1998). Foveal dysfunction and central visual field loss in glaucoma. Arch. Ophthalmol..

[28] Hood DC, Raza AS, de Moraes CGV, Liebmann JM, Ritch R (2013). Glaucomatous damage of the macula. Prog. Retin. Eye Res..

[29] Mwanza J-C, Kim HY, Budenz DL (2015). Residual and dynamic range of retinal nerve fiber layer thickness in glaucoma: Comparison of three OCT platforms. Invest. Ophthalmol. Vis. Sci..

[30] Mwanza J-C, Budenz DL, Warren JL (2015). Retinal nerve fibre layer thickness floor and corresponding functional loss in glaucoma. Br. J. Ophthalmol..

[31] Hood DC, Kardon RH (2007). A framework for comparing structural and functional measures of glaucomatous damage. Prog. Retin. Eye Res..

[32] Hood DC, Anderson SC, Wall M, Kardon RH (2007). Structure versus function in glaucoma: An application of a linear model. Invest. Ophthalmol. Vis. Sci..

[33] Kim JH, Lee HS, Kim NR, Seong GJ, Kim CY (2014). Relationship between visual acuity and retinal structures measured by spectral domain optical coherence tomography in patients with open-angle glaucoma. Invest. Ophthalmol. Vis. Sci..

[34] Bambo MP, Güerri N, Ferrandez B (2017). Evaluation of the macular ganglion cell-inner plexiform layer and the circumpapillary retinal nerve fiber layer in early to severe stages of glaucoma: Correlation with central visual function and visual field indexes. Ophthalmic Res..

[35] Na JH, Kook MS, Lee Y, Baek S (2012). Structure-function relationship of the macular visual field sensitivity and the ganglion cell complex thickness in glaucoma. Invest. Ophthalmol. Vis. Sci..

[36] Ghahari E, Bowd C, Zangwill LM (2019). Association of macular and circumpapillary microvasculature with visual field sensitivity in advanced glaucoma. Am. J. Ophthalmol..

[37] Liu L, Edmunds B, Takusagawa HL (2019). Projection-resolved optical coherence tomography angiography of the peripapillary retina in glaucoma. Am. J. Ophthalmol..

[38] Chen HS-L, Liu C-H, Wu W-C, Tseng H-J, Lee Y-S (2017). Optical coherence tomography angiography of the superficial microvasculature in the macular and peripapillary areas in glaucomatous and healthy eyes. Investig. Ophthalmol. Vis. Sci..

[39] Yarmohammadi A, Zangwill LM, Diniz-Filho A (2016). Optical coherence tomography angiography vessel density in healthy, glaucoma suspect, and glaucoma eyes. Investig. Ophthalmol. Vis. Sci..

[40] Shoji T, Zangwill LM, Akagi T (2017). Progressive macula vessel density loss in primary open-angle glaucoma: A longitudinal study. Am. J. Ophthalmol..

[41] Hormel TT, Jia Y, Jian Y (2021). Plexus-specific retinal vascular anatomy and pathologies as seen by projection-resolved optical coherence tomographic angiography. Prog. Retin. Eye Res..

[42] Rutkowski P, May CA (2016). Nutrition and vascular supply of retinal ganglion cells during human development. Front. Neurol..

[43] Akil H, Chopra V, Al-Sheikh M (2018). Swept-source OCT angiography imaging of the macular capillary network in glaucoma. Br. J. Ophthalmol..

[44] Fard MA, Fakhraee G, Ghahvechian H, Sahraian A, Moghimi S, Ritch R (2020). Macular vascularity in ischemic optic neuropathy compared to glaucoma by projection-resolved optical coherence tomography angiography. Am. J. Ophthalmol..

[45] Yoshikawa Y, Shoji T, Kanno J (2020). Glaucomatous vertical vessel density asymmetry of the temporal raphe detected with optical coherence tomography angiography. Sci. Rep..

[46] Park SC, Kung Y, Su D (2013). Parafoveal scotoma progression in glaucoma: Humphrey 10–2 versus 24–2 visual field analysis. Ophthalmology.

[47] Phu J, Kalloniatis M (2020). Ability of 24–2C and 24–2 grids to identify central visual field defects and structure-function concordance in glaucoma and suspects. Am. J. Ophthalmol..

[48] Chakravarti T, Moghadam M, Proudfoot JA, Weinreb RN, Bowd C, Zangwill LM (2021). Agreement between 10–2 and 24–2C visual field test protocols for detecting glaucomatous central visual field defects. J. Glaucoma.

[49] Peracha M, Hughes B, Tannir J (2013). Assessing the reliability of humphrey visual field testing in an urban population. Invest. Ophthalmol. Vis. Sci..

[50] Talisa E, Chin AT, Bonini Filho MA (2015). Detection of microvascular changes in eyes of patients with diabetes but not clinical diabetic retinopathy using optical coherence tomography angiography. Retina.

[51] Sun C, Ladores C, Hong J (2020). Systemic hypertension associated retinal microvascular changes can be detected with optical coherence tomography angiography. Sci. Rep..

[52] Quaranta L, Gandolfo F, Turano R (2006). Effects of topical hypotensive drugs on circadian IOP, blood pressure, and calculated diastolic ocular perfusion pressure in patients with glaucoma. Invest. Ophthalmol. Vis. Sci..

[53] Yu S, Frueh BE, Steinmair D (2018). Cataract significantly influences quantitative measurements on swept-source optical coherence tomography angiography imaging. PLoS ONE.

